# Fertility preservation in endocrine responsive breast cancer: data and prejudices

**DOI:** 10.3332/ecancer.2020.1157

**Published:** 2020-12-18

**Authors:** Barbara Buonomoa, Fedro A. Peccatorib

**Affiliations:** Fertility and Procreation Unit in Oncology, Gynecologic Oncology Program, European Institute of Oncology IRCCS, Via Ripamonti 435, 20141 Milan, Italy; a https://orcid.org/0000-0001-9921-4639; b https://orcid.org/0000-0001-8227-8740

**Keywords:** oestrogen receptor, breast cancer, fertility preservation, hormonal stimulation, ovarian stimulation

## Abstract

Even if current guidelines suggest an early referral of young breast cancer (BC) patients to fertility preservation counselling, physicians still lack knowledge about the different available strategies. Hormonal stimulation to harvest mature oocytes is considered unsafe by many oncologists and experts in reproductive medicine, particularly in the setting of oestrogen receptor-positive BC. The aim of this mini-review is to provide an overview on the available data about this topic in order to clarify potential misunderstandings and to highlight the new trends in the oncofertility field with their pros and limitations.

The European Society of Medical Oncology [1], the American Society of Reproductive Medicine [2] and the American Society of Clinical Oncology [3] recommend to discuss the potential risk of iatrogenic premature ovarian failure and infertility with all young cancer patients before starting anticancer treatments [1–5]. Breast cancer (BC) represents the most frequent oncological diagnosis in women during reproductive years [4]. Unfortunately, BC survivors have a low chance of pregnancy after diagnosis compared to their normal counterpart [6], especially when adjuvant endocrine therapy is prescribed [7], even if pregnancy is considered an important goal for many young patients [4]. The potential impact of pregnancy on the risk of recurrence is another major concern [8]. To date, several studies have demonstrated that pregnancy is safe in women with a history of BC following adequate treatment and follow-up and that it does not increase the risk of recurrence, even in patients with hormone receptor-positive disease [8, 9] and/or carriers of germline BRCA mutations [10].

Controlled ovarian stimulation (COS) and subsequent oocyte/embryo freezing are the gold standard of fertility preservation (FP) in this setting [1–5]. Nonetheless, when a focused survey was administered to physicians attending two major BC conferences, 37% of the respondents (101/273) reported that they had never consulted the international guidelines about FP and 22.3% considered COS not safe in the setting of oestrogen receptor (ER)-positive BC [11]. Two major concerns were raised: the potential delay in the start of chemotherapy (CHT) and the possible enhancing effects of rising of oestradiol (E2) levels during OS on BC growth/recurrence.

Are these issues really clinically relevant?

## Chemotherapy delay

Several studies have showed no detrimental effects on BC recurrence or survival if CHT was delayed until 12 weeks after surgery, particularly in ER-positive, early stage BC patients [12, 13]. Nowadays, thanks to an early referral to fertility specialists and the need of no more than 2 weeks for COS and oocyte retrieval, oocytes/embryos cryopreservation can be safely completed without significant CHT delay [1, 14]. The possibility of starting stimulation at any day of the menstrual cycle (random start protocol) [15] and the option of a double stimulation make this procedure feasible and effective, allowing to optimise the number of retrieved oocytes without affecting BC prognosis [1, 16, 17].

## Detrimental effects of high oestrogen levels

Up to now, there is no evidence that COS promotes BC growth [18]. A systematic review by Rodgers *et al* [19] did not show any association between COS and an increased risk of BC relapse. Oktay *et al* [20–22] developed a modified OS protocol based on the concurrent use of the aromatase inhibitor letrozole and gonadotrophins, in order to maintain oestrogen levels similar to those reached in unstimulated cycle without affecting oocyte and embryo yield. The same group conducted a prospective non-randomised study which compared 79 BC patients who underwent COS with letrozole and gonadotrophins and 136 patients that did not perform FP as controls [23]. Most of the patients in the COS group (64/79; 81%) had a diagnosis of ER-positive BC. The median follow-up after adjuvant CHT was 23.4 months in the treatment group and 33.05 months in the control one. The recurrence risk was not increased compared with controls [23]. Subsequently, a prospective non-randomised study by Kim *et al* [24] confirmed this data within a longer follow-up of 5 years after diagnosis. They also reported no difference in survival among women with BRCA mutation who underwent OS [24].

More recently, Letourneau *et al* [25] compared the oncological outcome of 207 BC patients who underwent COS for FP and 122 who did not. With a median follow-up of 43 months, no impairment of DFS was reported in the FP group, even among women with ER-positive BC (DFS: HR, 0.4; 95% CI, 0.1–1.6) [25]. Authors acknowledge the retrospective nature of the study, but conclude that FP appears unlikely to affect DFS, even in the setting of neoadjuvant CHT (NACT) (in which the tumour is still present during FP). To date, NACT has gained popularity as it improves the rate of breast conserving surgery and allows *in vivo* tumour response to adapt the subsequent adjuvant treatment [26]. Kim *et al* [24] reported only one recurrence among 14 BC patients who underwent COS before surgery (7%) and 5 among 106 patients who underwent the same treatment after surgery (4%) (*p* = 0.47). The relapse-free survival rate was not statistically significantly different between pre- and post-surgery groups (*p* = 0.44) [24]. Chien *et al* [27] conducted a retrospective case–control study to evaluate the impact of COS on the time of the initiation of NACT. The mean time from diagnosis to NACT was 39.8 days versus 40.9 days (*p* = 0.75), and the median time was 41.5 days versus 35.5 days (*p* = 0.50) in the 34 patients who underwent COS versus the 48 control patients, respectively. Thus, patients who underwent COS before NACT had a delay of approximately 1 week compared to control patients, which could hardly impact prognosis [27]. Nonetheless, data about the safety of this approach focusing on ER-positive BC are still limited and long-term follow-up is needed. In case of very aggressive tumours, where CHT cannot be postponed for 2–3 weeks, ovarian tissue cryopreservation (OTC) may be considered [28].

A prospective multicentre study conducted by Marklund *et al* [29] compared different FP strategies among 610 young BC patients who underwent CHT. Of these, 401 performed COS; after a mean follow-up of 6.3 years, no differences in terms of survival were reported between patients who underwent COS and those who did not, between those who used letrozole in the COS protocol or those who used a ‘random start’ protocol. Hence, all the available data support safety and efficacy of COS in BC patients, whenever it is possible according to their age, ovarian reserve and tumour characteristics [18–27, 29].

## Alternative options

Novel strategies have been developed to avoid hormonal stimulation in BC patients: harvesting ovarian tissue with subsequent cryopreservation, harvesting immature oocytes without COS (*in vitro* maturation—IVM) or using gonadotrophin-releasing hormone analogues (GnRHa) as medical gonadoprotection.

Over 130 live births have been reported after reimplantation of ovarian tissue [2, 5, 30–41], with live birth rates exceeding 35% [5]. Nonetheless, the possible advantages of OTC should not be overemphasised, as there is a theoretical possibility of re-implanting malignant cells [42], and the procedure should not be offered to patients harbouring BRCA mutation, for the increased risk of subsequent ovarian cancer [2, 43].

Grynberg *et al* [44] reported the first live birth using IVM in a young woman with a diagnosis of ER-positive BC. Even if progresses in sustaining *in vitro* growth of human oocytes have been made, the pregnancy outcomes after IVM are still suboptimal, with lower implantation rates as compared with embryos obtained from mature oocytes [45, 46].

According to current guidelines, the ovarian suppression with GnRHa during CHT does not represent an option of FP but a strategy to reduce the detrimental impact of cytotoxic drugs on ovarian reserve [47] as data on post-treatment pregnancies remain limited [48–50]. While medical gonadoprotection should proposed to all premenopausal patients concerned about the risk of premature ovarian insufficiency (POI), patients interested in future pregnancies should be always offered oocyte/embryo cryopreservation. Further studies are needed about GnRHa role in the setting of BRCA-mutated patients [1].

To date, evidence on long-term reproductive outcomes after BC treatment is still scarce. A recent Swedish cohort study indicates that a successful pregnancy after BC is possible both in women who underwent FP and who did not [51]. Noteworthy, the authors showed that FP is associated with significantly higher rates of post-BC live births, without any detrimental impact on survival outcomes during a mean follow-up of 5.2 years [51]. Nonetheless, FP options still have several limitations, such as a limited awareness among both the general population and also medical oncologists and the lack of ‘fast-track’ referral pathway. We have also to consider the psychological pressure added by an oncofertility counselling at the time of cancer diagnosis. Even if several countries provide FP procedures entirely with no costs for the patients, there are still realities with no coverage of costs or with a very limited refund depending on patient characteristics (disease, prognosis, age) or on the number of treatments (number of cycles, first child) [52]. Age is the most crucial factor swaying the success rate of FP: the efficacy of oocyte/embryo cryopreservation is strictly related to the number of mature oocytes retrieved, that is age-dependent, dropping precipitously after 35 years of age [53, 54]. Moreover, the effectiveness of COS may be negatively impacted by BRCA status, considering the emerging evidence of a diminished ovarian reserve and a poor response to ovarian stimulation in *BRCA1/2*-mutation carriers [55, 56].

## Conclusion

In conclusion, a timely COS for FP remains the first choice for young BC patients interested in FP, also if they have ER-positive tumours. Modified COS protocols with letrozole combined with gonadotrophins could increase safety and it should be recommended [18, 19, 29, 57, 58]. However, the decision to adopt this strategy should be balanced and multidisciplinary. Considering the new emerging evidences, every patient should be counselled about the gonadotoxicity of the proposed treatments and the available strategies to prevent POI and preserve fertility but the possibility to access must be evaluated case by case, assessing the risk of gonadotoxicity, ovarian reserve at baseline and cost-effectiveness. It is of paramount importance to ponder for each patient the most appropriate technique for FP and discuss reproductive outcomes relying on data and not on prejudices.

## Conflicts of interest

The authors declare that they have no conflicts of interest.

## Funding statement

No funding was received for this specific research.

## Authors’ contributions

All authors listed have contributed sufficiently to the writing and/or critical revision of the paper; they have approved the submitted final version.

## References

[1] Lambertini M, Peccatori FA, Demeestere I (2020). Fertility preservation and post-treatment pregnancies in post-pubertal cancer patients: ESMO Clinical Practice Guideline. Ann Oncol.

[2] Practice Committee of the American Society for Reproductive Medicine (2019). Electronic address: asrm@asrm.org. Fertility preservation in patients undergoing gonadotoxic therapy or gonadectomy: a committee opinion. Fertil Steril.

[3] Loren AW, Mangu PB, Beck LN (2013). Fertility preservation for patients with cancer: American Society of Clinical Oncology clinical practice guideline update. J Clin Oncol.

[4] Paluch-Shimon S, Cardoso F, Partridge AH (2020). ESO-ESMO 4th International Consensus Guidelines for Breast Cancer in Young Women (BCY4). Ann Oncol.

[5] Donnez J, Dolmans MM (2018). Fertility preservation in women. N Engl J Med.

[6] Anderson RA, Brewster DH, Wood R (2018). The impact of cancer on subsequent chance of pregnancy: a population-based analysis. Hum Reprod.

[7] Shandley LM, Spencer JB, Fothergill A (2017). Impact of tamoxifen therapy on fertility in breast cancer survivors. Fertil Steril.

[8] Azim HA, Kroman N, Paesmans M (2013). Prognostic impact of pregnancy after breast cancer according to estrogen receptor status: a multicenter retrospective study. J Clin Oncol.

[9] Lambertini M, Kroman N, Ameye L (2018). Long-term safety of pregnancy following breast cancer according to estrogen receptor status. J Natl Cancer Inst.

[10] Lambertini M, Ameye L, Hamy AS (2020). Pregnancy after breast cancer in patients with germline BRCA mutations. J Clin Oncol.

[11] Lambertini M, Di Maio M, Pagani O (2018). The BCY3/BCC 2017 survey on physicians’ knowledge, attitudes and practice towards fertility and pregnancy-related issues in young breast cancer patients. Breast.

[12] Cold S, Düring M, Ewertz M (2005). Does timing of adjuvant chemotherapy influence the prognosis after early breast cancer? Results of the Danish Breast Cancer Cooperative Group (DBCG). Br J Cancer.

[13] Lohrisch C, Paltiel C, Gelmon K (2006). Impact on survival time from definitive surgery to initiation of adjuvant chemotherapy for early-stage breast cancer. J Clin Oncol.

[14] Lee S, Heytens E, Moy F (2011). Determinants of access to fertility preservation in women with breast cancer. Fertil Steril.

[15] Danis RB, Pereira N, Elias RT (2017). Random start ovarian stimulation for oocyte or embryo cryopreservation in women desiring fertility preservation prior to gonadotoxic cancer therapy. Curr Pharm Biotechnol.

[16] Yager JD, Davidson N (2006). Estrogen carcinogenesis in breast cancer. N Engl J Med.

[17] Muñoz E, González N, Muñoz L (2015). Ovarian stimulation in patients with breast cancer. Ecancermedicalscience.

[18] Dahhan T, Balkenende E, van Wely M (2013). Tamoxifen or letrozole versus standard methods for women with estrogen-receptor positive breast cancer undergoing oocyte or embryo cryopreservation in assisted reproduction. Cochrane Database Syst Rev.

[19] Rodgers RJ, Reid GD, Koch J (2017). The safety and efficacy of controlled ovarian hyperstimulation for fertility preservation in women with early breast cancer: a systematic review. Hum Reprod.

[20] Oktay K, Buyuk E, Libertella N (2005). Fertility preservation in breast cancer patients: A prospective controlled comparison of ovarian stimulation with tamoxifen and letrozole for embryo cryopreservation. J Clin Oncol.

[21] Oktay K (2005). Further evidence on the safety and success of ovarian stimulation with letrozole and tamoxifen in breast cancer patients undergoing in vitro fertilization to cryopreserve their embryos for fertility preservation. J Clin Oncol.

[22] Azim A, Costantini-Ferrando M, Oktay K (2007). Relative potencies of anastrozole and letrozole to sup- press estradiol in breast cancer patients undergoing ovarian stimulation before in vitro fertilization. J Clin Endocrinol Metab.

[23] Azim AA, Costantini-Ferrando M, Oktay K (2008). Safety of fertility preservation by ovarian stimulation with letrozole and gonadotropins in patients with breast cancer: a prospective controlled study. J Clin Oncol.

[24] Kim J, Turan V, Oktay K (2016). Long-term safety of letrozole and gonadotropin stimulation for fertility preservation in women with breast cancer. J Clin Endocrinol Metab.

[25] Letourneau JM, Wald K, Sinha N (2020). Fertility preservation before breast cancer treatment appears unlikely to affect disease-free survival at a median follow-up of 43 months after fertility-preservation consultation. Cancer.

[26] Caparica R, Lambertini M, Pondé N (2019). Post-neoadjuvant treatment and the management of residual disease in breast cancer: state of the art and perspectives. Ther Adv Med Oncol.

[27] Chien AJ, Chambers J, Mcauley F (2017). Fertility preservation with ovarian stimulation and time to treatment in women with stage II-III breast cancer receiving neoadjuvant therapy. Breast Cancer Res Treat.

[28] Arecco L, Perachino M, Damassi A (2020). Burning questions in the oncofertility counseling of young breast cancer patients. Breast Cancer (Auckl).

[29] Marklund A, Eloranta S, Wikander I (2020). Efficacy and safety of controlled ovarian stimulation using GnRH antagonist protocols for emergency fertility preservation in young women with breast cancer - a prospective nationwide Swedish multicenter study. Hum Reprod.

[30] Silber S, Kagawa N, Kuwayama M (2010). Duration of fertility after fresh and frozen ovary transplantation. Fertil Steril.

[31] Silber SJ, Gosden RG (2007). Ovarian transplantation in a series of monozygotic twins discordant for ovarian failure N Engl J Med.

[32] Donnez J, Silber S, Andersen CY (2011). Children born after autotransplantation of cryopreserved ovarian tissue. a review of 13 live births. Ann Med.

[33] Oktay K (2002). Evidence for limiting ovarian tissue harvesting for the purpose of transplantation to women younger than 40 years of age. J Clin Endocrinol Metab.

[34] Donnez J, Dolmans MM, Demylle D (2004). Live-birth after orthotopic transplantation of cryopreserved ovarian tissue. Lancet.

[35] Donnez J, Squiflet J, Jadoul P (2011). Pregnancy and live birth after autotransplantation of frozen-thawed ovarian tissue in a patient with metastatic disease undergoing chemotherapy and hematopoietic stem cell transplantation. Fertil Steril.

[36] Meirow D, Levron J, Eldar-Geva T (2005). Pregnancy after transplantation of cryopreserved ovarian tissue in a patient with ovarian failure after chemotherapy. N Engl J Med.

[37] Ernst E, Bergholdt S, Jorgensen JS (2010). The first woman to give birth to two children following transplantation of frozen/thawed ovarian tissue. Hum Reprod.

[38] Sanchez-Serrano M, Crespo J, Mirabet V (2010). Twins born after transplantation of ovarian cortical tissue and oocyte vitrification. Fertil Steril.

[39] Andersen CY, Rosendahl M, Byskov AG (2008). Two successful pregnancies following autotransplantation of frozen/thawed ovarian tissue. Hum Reprod.

[40] Roux C, Amiot C, Agnani G (2010). Live birth after ovarian tissue autograft in a patient with sickle cell disease treated by allogeneic bone marrow transplantation. Fertil Steril.

[41] Dittrich R, Lotz L, Keck G (2012). Live birth after ovarian tissue autotransplantation following overnight transportation before cryopreservation. Fertil Steril.

[42] Meirow D, Hardan I, Dor J (2008). Searching for evidence of disease and malignant cell contamination in ovarian tissue stored from hematologic cancer patients. Hum Reprod.

[43] Peccatori FA, Mangili G, Bergamini A (2018). Fertility preservation in women harboring deleterious BRCA mutations: ready for prime time?. Hum Reprod.

[44] Grynberg M, Mayeur Le Bras A, Hesters L (2020). First birth achieved after fertility preservation using vitrification of in vitro matured oocytes in a woman with breast cancer. Ann Oncol.

[45] Zhang XY, Ata B, Son WY (2010). Chromosome abnormality rates in human embryos obtained from in-vitro maturation and IVF treatment cycles. Reprod Biomed Online.

[46] Sermondade N, Sonigo C, Sifer C (2019). Serum antimüllerian hormone is associated with the number of oocytes matured in vitro and with primordial follicle density in candidates for fertility preservation. Fertil Steril.

[47] Lambertini M, Richard F, Nguyen B (2019). Ovarian function and fertility preservation in breast cancer: should gonadotropin-releasing hormone agonist be administered to all premenopausal patients receiving chemotherapy?. Clin Med Insights Reprod Health.

[48] Moore HC, Unger JM, Phillips KA (2015). Goserelin for ovarian protection during breast-cancer adjuvant chemotherapy. N Engl J Med.

[49] Lambertini M, Ceppi M, Poggio F (2015). Ovarian suppression using luteinizing hormone-releasing hormone agonists during chemotherapy to preserve ovarian function and fertility of breast cancer patients: a meta-analysis of randomized studies. Ann Oncol.

[50] Lambertini M, Moore HCF, Leonard RCF (2018). Gonadotropin-releasing hormone agonists during chemotherapy for preservation of ovarian function and fertility in premenopausal patients with early breast cancer: a systematic review and meta-analysis of individual patient-level data. J Clin Oncol.

[51] Marklund A, Lundberg FE, Eloranta S (2020). Reproductive outcomes after breast cancer in women with vs without fertility preservation. JAMA Oncol.

[52] Anderson RA, Amant F, ESHRE Guideline Group on Female Fertility Preservation (2020). ESHRE guideline: female fertility preservation. Hum Reprod Open.

[53] Cimadomo D, Fabozzi G, Vaiarelli A (2018). Impact of maternal age on oocyte and embryo competence. Front Endocrinol (Lausanne).

[54] Law YJ, Zhang N, Venetis CA (2019). The number of oocytes associated with maximum cumulative live birth rates per aspiration depends on female age: a population study of 221 221 treatment cycles. Hum Reprod.

[55] Titus S, Li F, Stobezki R (2013). Impairment of BRCA1-related DNA double-strand break repair leads to ovarian aging in mice and humans. Sci Transl Med.

[56] Porcu E, Cillo GM, Cipriani L (2020). Impact of BRCA1 and BRCA2 mutations on ovarian reserve and fertility preservation outcomes in young women with breast cancer. J Assist Reprod Genet.

[57] Oktay K, Buyuk E, Rodriguez-Wallberg KA (2010). In vitro maturation improves oocyte or embryo cryopreservation outcome in breast cancer patients undergoing ovarian stimulation for fertility preservation. Reprod Biomed Online.

[58] Reddy J, Turan V, Bedoschi G (2014). Triggering final oocyte maturation with gonadotropin-releasing hormone agonist (GnRHa) versus human chorionic gonadotropin (hCG) in breast cancer patients undergoing fertility preservation: an extended experience. J Assist Reprod Genet.

